# Measles and public health: an integrative approach

**DOI:** 10.1186/s13062-025-00693-0

**Published:** 2025-10-14

**Authors:** Francesco Branda, Marta Giovanetti, Nicola Petrosillo, Mohamed Mustaf Ahmed, Maria Perra, Daria Sanna, Giancarlo Ceccarelli, Massimo Ciccozzi, Enrico Bucci, Fabio Scarpa

**Affiliations:** 1 Unit of Medical Statistics and Molecular Epidemiology, Università Campus Bio-Medico di Roma, Via Álvaro del Portillo, 21, 00128 Rome, RM Italy; 2 Genomics, AI, Bioinformatics, Infectious Diseases, Epidemiology Group (GABIE), Rome, Italy; 3 Department of Science and Technologies for Sustainable Development and One Health, Università Campus Bio-Medico di Roma, Via Álvaro del Portillo, 21, 00128 Rome, RM Italy; 4 Instituto Rene Rachou, Fundação Oswaldo Cruz (FIOCRUZ), Av. Augusto de Lima, 1715, Belo Horizonte, MG 30190-002 Brazil; 5 Infection Prevention & Control/Infectious Disease Service, Fondazione Policlinico Universitario Campus Bio-Medico, 00127 Rome, Italy; 6 Faculty of Medicine and Health Sciences, SIMAD University, Mogadishu, Somalia; 7 Department of Biomedical Sciences, University of Sassari, Viale San Pietro, 43, 07100 Sassari, SS Italy; 8 Department of Public Health and Infectious Diseases, University of Rome Sapienza, Piazzale Aldo Moro, 5, 00185 Rome, RM Italy; 9 Department of Biology, Sbarro Institute for Cancer Research and Molecular Medicine, Temple University, 1900 North 12th Street, Philadelphia, PA 19122 USA

**Keywords:** Measles, Outbreak dynamics, Spatial modeling, Public health response, Genomic surveillance, Phylogenomic analysis, Genetics

## Abstract

**Background:**

Measles, once considered under control in many high-income countries, has experienced a notable resurgence in recent years due to declining vaccination rates, increased vaccine hesitancy, and gaps in public health preparedness. This study provides an overview of the current measles outbreaks in two socio-culturally distinct realities, both facing a challenging epidemiological situation, i.e., the Region of the Americas and Italy, the European country most impacted after Romania. The aim is to understand transmission dynamics and identify factors contributing to outbreak severity.

**Results:**

Epidemiological data show that Canada experienced an unprecedented increase in measles incidence, particularly in Ontario and Alberta, where spatial modelling revealed relative risks greater than 30 in high burden areas (i.e., the estimated likelihood of measles occurrence in these areas was more than 30 times higher than the national average, based on Bayesian spatial modeling). In Mexico, the epidemic was highly localised, with over 90% of cases and all but one death concentrated in the state of Chihuahua. In the United States, 89% of cases were linked to epidemic outbreaks, with Texas showing significant spatial clustering and daily growth rates of over 4% in high-risk counties. In Italy, the 2024 outbreak marked a significant increase in measles cases compared to previous years, primarily affecting unvaccinated individuals. Over 50% of those affected required hospitalization, and major urban regions such as Lazio and Lombardy experienced sustained transmission. An initial phase of exponential growth (66% monthly) was followed by a plateau, with no significant decline observed, underlining delays in containment and persistent immune deficiencies. From genetic point of view, the study revealed the predominance of genotype D8, known for sustained global circulation, suggesting a single transmission chain behind the recent outbreaks. Phylogenetic analysis showed no significant intra-genotypic diversification, suggesting that the outbreak likely originated from a single introduction event followed by rapid, localized transmission. This limited genetic variation is consistent with a short transmission window and the absence of strong evolutionary pressure.

**Conclusion:**

The outbreaks in the United States and Italy, despite differences in healthcare systems and sociopolitical contexts, reveal common underlying issues. In the U.S., the epidemic was characterized by clusters of unvaccinated individuals in certain communities, while Italy faced challenges due to gaps in routine immunization programs and delays in responding to the outbreak. Both outbreaks illustrate the devastating impact of under-vaccination, inadequate surveillance, and the spread of misinformation on public health. Our results contribute to a deeper understanding of the dynamics of measles recurrence, providing a solid basis for science-based prevention and control measures. Furthermore, the study emphasises the importance of continuous and integrated surveillance to detect emerging or divergent strains at an early stage.

**Clinical trial number:**

Not applicable.

## Background

Measles, caused by the *Measles morbillivirus*, is one of the most contagious viral diseases known, with a basic reproduction number (R$_0$) ranging from 12 to 18—making it more transmissible than other common infectious diseases, including influenza and SARS-CoV-2 [1]. Transmission occurs primarily through respiratory droplets and direct contact; the virus can also remain airborne or survive on surfaces for up to two hours. Clinical manifestations include high fever, cough, and rash, and can lead to severe complications such as pneumonia, encephalitis, and subacute sclerosing panencephalitis (SSPE), particularly among young children and immunocompromised individuals [2, 3]. Before the introduction of vaccination programs, measles caused an estimated 2.6 million deaths globally each year, representing a major burden to health systems and a significant threat to child survival [4]. The widespread introduction of measles-containing vaccines (MCVs)—including the widely used MMR (measles-mumps-rubella) formulation—in the 1960s led to a dramatic reduction in both incidence and mortality. Through sustained immunization efforts, many high-income countries achieved measles elimination, defined as the absence of endemic transmission for more than 12 months in the presence of a well-performing surveillance system. For instance, the U.S. declared measles eliminated in 2000 [5]. Italy had temporarily achieved elimination status prior to 2017, following aggressive vaccination strategies, but this status was lost the same year due to a major outbreak driven by declining vaccine coverage [6]. However, despite these initial successes, recent years have witnessed a troubling global resurgence of measles, even in countries with historically strong healthcare infrastructures. This resurgence has been driven by a complex interplay of factors. Declining vaccination coverage—often falling below the critical herd immunity threshold of 95% has been observed in multiple regions, fueled in part by growing vaccine hesitancy and misinformation campaigns, particularly via social media [7, 8]. Additional contributing factors include systemic inequities in healthcare access, under-resourced public health systems, and disruptions to routine immunization services—exacerbated by the COVID-19 pandemic—which have created immunity gaps, especially among vulnerable and marginalized populations [9]. The global resurgence of measles highlights systemic gaps in both surveillance and immunization programs, as shown by recent work analyzing spatiotemporal trends and the implications of declining coverage in multiple WHO regions [10]. In 2020 alone, the World Health Organization (WHO) reported that over 22 million infants missed their first dose of the measles vaccine, marking the largest increase in two decades [11]. Notably, Europe and the U.S. have not been immune to these trends. Italy has experienced repeated outbreaks over the past decade, with more than 4,000 cases reported in 2017 alone, largely concentrated in regions with low MMR uptake [6]. Similarly, in the U.S., despite national elimination status, localized outbreaks have occurred in under-vaccinated communities—often initiated by imported cases and amplified by social, cultural, or religious barriers to vaccination [12]. In this context, the recent measles outbreaks in Italy and Americas offer valuable opportunities to examine the complex dynamics of measles re-emergence in high-income settings. Although both occurred in relatively well-resourced healthcare environments, the underlying social, political, and public health conditions differed significantly, providing a comparative lens through which to assess outbreak risk factors, vaccination trends, and response strategies. Based on these two case studies, the paper analyzes the epidemiological profiles of the outbreaks, assess the effectiveness of national and local public health interventions, and explore broader implications for global measles control. By identifying common vulnerabilities and divergent policy outcomes, we aim to inform the development of more resilient and equity-oriented immunization programs and outbreak preparedness strategies. Specifically, Sect. 2 describes the materials and methods used in this study, including the epidemiological data sources, statistical analyses, and genetic sequencing approaches. Section 3 presents the results and discussion, where we analyze the measles outbreaks of the Region of the Americas and Italy, highlighting the factors that contributed to their resurgence. This section also includes an in-depth exploration of the genetic diversity of the measles virus, based on phylogenetic analyses, and evaluating the implications of our findings for global measles control, focusing on the effectiveness of public health interventions, the role of vaccination campaigns, and the need for strengthened surveillance systems. Finally, Sect. 4 concludes the work, providing conclusions and recommendations for more resilient, equity-driven immunization programs and enhanced outbreak preparedness strategies.

## Methods

### Epidemiology analyses

Epidemiological data on measles cases used in this study for the Region of Americas were extracted from the Measles Tracker platform [13], a near-real-time data hub for measles surveillance that compiles official case reports from ministries of health, PAHO, and other verified national sources. The platform ensures data standardization through multi-level quality control processes, including temporal consistency checks, duplicate detection, and geographic validation of reported cases. For Italy, we used case data from the official monthly epidemiological bulletins in PDF format [14] published by the Istituto Superiore di Sanità (ISS). These reports included detailed counts of confirmed cases, hospitalizations, deaths, and information on comorbidities, along with age-stratified and sex-disaggregated data, enabling more granular analyses of the epidemic’s progression across demographic groups. To enhance data accessibility, we developed a real-time data portal in collaboration with Il Sole 24 Ore (https://lab24.ilsole24ore.com/morbillo-italia-dati-iss/, accessed on July 10, 2025), which aggregates and continuously updates measles statistics.

Data preprocessing and statistical analyses were performed using the R programming language (version 4.5.1) within the RStudio environment (version 2025.05.1). The analysis relied on a specialized ecosystem of R packages, including dplyr for data manipulation, ggplot2 for visualization, and lubridate for date handling. Geographic distributions of cases were visualized using sf (version 1.0-14) for spatial data handling, rnaturalearth for map data, and tigris for US geographic boundaries. Maps were created using the WGS84 coordinate reference system with custom choropleth visualizations showing cumulative case distributions at state/province and municipality levels. To estimate spatial variation in measles risk, we employed a Bayesian spatial modeling approach based on Stochastic Partial Differential Equations (SPDE) within the Integrated Nested Laplace Approximation (INLA) framework. Spatial random effects were modeled using a Matérn covariance structure, with computations performed via the INLA and inla.spde2.matern functions. A triangulated mesh over the study area was generated to discretize the spatial domain, and a projection matrix was created to link observed data locations to mesh nodes.

The model assumed a Poisson likelihood for the observed measles case counts:

$$y_i \sim \text{Poisson}(\lambda_i)$$

where $y_i$ is the observed number of measles cases in the $i$-th spatial unit, and $\lambda_i$ is the expected number of cases, which is modeled as:

$$\lambda_i = \exp{\left(\beta_0 + X_i \beta + z_i\right)}$$

Here:

- $\beta_0$ is the intercept,
- $X_i$ are the covariates (such as vaccination coverage, socio-economic factors),
- $X_i$ are the covariates (such as vaccination coverage, socio-economic factors),
- $\beta$ are the regression coefficients for the covariates, and
- $z_i$ represents the spatial random effect, modeled as a realization from a Gaussian process with a Matérn covariance function:

$$\text{Cov}(z_i, z_j) = \sigma^2 \frac{\Gamma\left(\nu + \frac{d}{2}\right)}{2^\nu \Gamma(\nu)} \left( \frac{|x_i - x_j|}{\phi} \right)^\nu K_\nu\left( \frac{|x_i - x_j|}{\phi} \right)$$

where:

- $| \mathbf{x}_i - \mathbf{x}_j |$ is the distance between the spatial locations $\mathbf{x}_i$ and $\mathbf{x}_j$,
- $\sigma^2$ is the spatial variance,
- $\phi$ is the range parameter,
- $\nu$ is the smoothness parameter, and
- $K_\nu(\cdot)$ is the modified Bessel function of the second kind, which defines the smoothness of the process.

Posterior summaries of the spatial field were used to compute relative risk estimates (risk scores), including posterior means and 95% credible intervals. These were exponentiated to interpret effects on the natural scale:

$$RR_i = \exp{\left(z_i\right)}$$

where $RR_i$ is the relative risk of measles for the $i$-th spatial unit. In addition to relative risk estimation, we also calculated the Area Under the Curve (AUC) as a metric for epidemic burden. The AUC is defined as the integral of the epidemic curve, representing the cumulative burden of cases over time:

$$\text{AUC} = \int_{0}^{T} \text{Cases}(t) \, dt$$

where $\text{Cases}(t)$ is the number of cases at time $t$, and $T$ is the time horizon of the epidemic. A higher AUC indicates a greater cumulative impact of the epidemic, considering both the magnitude of cases and the duration of the outbreak.

Model fit was assessed using Deviance Information Criterion (DIC) and Watanabe-Akaike Information Criterion (WAIC):

$$\text{DIC} = \bar{D} + p_D$$

$$\text{WAIC} = -2 \sum_{i} \log{\hat{p}(y_i)} + 2 \text{Var}\log{p}(y_i)$$

where $\bar{D}$ is the posterior mean of the deviance, and $p_D$ is the effective number of parameters. The WAIC includes both the model’s goodness-of-fit and the penalty for complexity. These metrics were applied to guide model selection and calibration. Specifically, we compared multiple candidate specifications of the spatial model, which differed in: (i) the choice of prior distributions for the spatial random effects, (ii) the structure of the Matérn covariance function, and (iii) the inclusion of covariates. For each specification, we computed DIC and WAIC values from the posterior samples. The final model was selected as the one minimizing both criteria, as lower values indicate better predictive accuracy penalized for model complexity. This approach ensured that the chosen model provided an optimal balance between goodness-of-fit and parsimony, avoiding overfitting while retaining adequate flexibility to capture spatial heterogeneity.

Moreover to fitting the spatial model, we calculated the variability ($\omega$), which quantifies the fluctuations in the epidemic trajectory. Variability is calculated as the standard deviation of weekly case counts:

$$\omega = \sqrt{\frac{1}{N} \sum_{i=1}^{N} (C_i - \mu)^2}$$

where $C_i$ is the number of cases in week $i$, $\mu$ is the mean number of cases over all weeks, and $N$ is the total number of weeks. A high value of $\omega$ indicates significant fluctuations in the weekly case counts, while a low $\omega$ suggests a more stable, predictable progression of the epidemic.

Based on the estimated risk scores, we applied k-means clustering to classify geographic units into low-, medium-, and high-risk clusters. The clustering was performed using the following optimization criterion:

$$J = \sum_{i=1}^{N} | z_i - C_k |^2$$

where $\mathbf{z}_i$ is the vector of spatial risk scores, and $\mathbf{C}_k$ is the centroid of the $k$-th cluster.

Finally, to assess the goodness-of-fit of the epidemic predictions, we calculated the coefficient of determination $R^2$ based on the correlation between observed and predicted case counts:

$$R^2 = \left( r \right)^2 = \left( \mathrm{cor}(y_\text{obs}, y_\text{pred}) \right)^2$$

where:

- $y_\text{obs}$ is the vector of observed measles case counts for the considered spatial unit or province,
- $y_\text{pred}$ is the vector of predicted measles case counts from the model,
- $r$ is the Pearson correlation coefficient between $y_\text{obs}$ and $y_\text{pred}$.

The $R^2$ value indicates the proportion of the observed variability explained by the model, with values close to 1 reflecting a very good fit.

Choropleth maps were generated to visualize spatial risk patterns, and county-level cluster labels were overlaid using centroids of spatial polygons. The approach enabled high-resolution identification of localized areas with elevated measles risk, complementing traditional surveillance outputs.

All analyses followed reproducible research principles with version-controlled R scripts. Data processing included date standardization, geographic grouping, and calculation of derived metrics such as cumulative case counts and spatially smoothed risk estimates. No personally identifiable information was used in this study, as all case data were aggregated at the administrative level in accordance with health data protection regulations.

### Genetic analyses

In order to obtain the most comprehensive picture possible, two different datasets were constructed for genetic analyses: one based on whole genomes, including 2,353 complete genomes sampled between January 1954 and May 2025, and another composed by isolates from 2021 to 2025 (n = 1,023), based solely on the N450 region (the last 450 nucleotides of the nucleoprotein gene coding region), since in 1998 it has been identified as the selection marker for standard molecular surveillance for measles [15, 16]. Datasets were built by downloading all available records from the NCBI Virus database [17]. Both datasets were aligned using the L-INS-I alignment strategy implemented in MAFFT version 7.471 [18]. Subsequent manual refinement was performed with Unipro UGENE v.35 [19]. To identify the best-fitting evolutionary model, jModelTest version 2.1.1 [20] was applied, using a maximum likelihood framework to optimize model selection. To contextualize the genetic variability of measles and describe the evolutionary trajectory over time within the broader evolutionary landscape, an initial phylogenomic analysis on the 2,353 complete genomes was performed. This investigation used global data and was performed with the nextstrain/ncov pipeline (https://github.com/nextstrain/ncov, accessed on 7 July 2025). Data were last updated on 10 June 2025. To investigate patterns of genetic variation and potential subclustering among the isolates, a Principal Coordinate Analysis (PCoA) was conducted using GenAlEx 6.5 [21]. This approach allowed the visualization of genetic relationships by representing dissimilarities derived from sequence variation. A pairwise p-distance matrix was generated from the genetic dataset through 1,000 iterations using the APE package in R [22]. The PCoA was subsequently performed using a covariance matrix approach, with prior data standardization applied to ensure comparability across variables. Bayesian Skyline Plots (BSPs) and Lineages Through Time (LTT) analyses were performed using the Bayesian inference approach implemented in BEAST version 1.10.4 [23], under the Bayesian Skyline coalescent model and an uncorrelated log-normal relaxed clock over 200 million generations. The graphs of BSP and LTT were generated with Tracer version 1.7.2 [24], which was also used to assess convergence and effective sample size (ESS) metrics. In all analyses, ESS values exceeded the recommended threshold of 200, supporting the reliability and robustness of the estimates.

## Results

### Epidemiological overview of measles outbreaks in the Region of the Americas

**Fig. 1:**
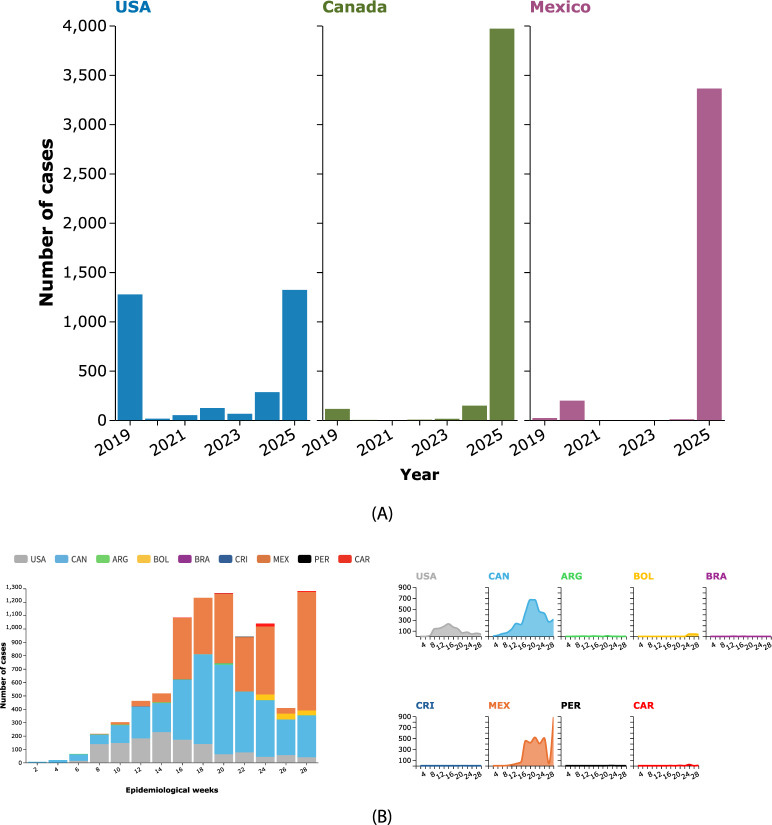
(**A**) Yearly measles cases in the most affected countries from 2019 to 2025. (**B**) Geographic distribution of confirmed measles cases by epidemiological week and by country/territory in the Region of the Americas, Weeks 1 to 28, 2025. Country codes are defined as follows: USA = United States; CAN = Canada; ARG = Argentina; BOL = Bolivia; BRA = Brazil; CRI = Costa Rica; MEX = Mexico; PER = Peru; CAR = Caribbean countries

In the last few years, according to the Pan American Health Organization (PAHO), the Region of the Americas experienced a sharp decrease in the overall number of measles cases, from 8,705 in 2019 to 464 in 2023. However, while the absolute case counts declined, their geographic distribution changed dramatically: the proportion of infections occurring in North America (United States, Canada, and Mexico) rose from 2.41% in 2019 to 98.15% in 2023. This indicates that the epidemic burden progressively shifted from South America—where most cases were initially concentrated—to North America. The current wave began in Texas in January 2025 and subsequently spread through networks of undervaccinated communities into Canada and Mexico (Fig. 1a), threatening the official measles-free status of all three countries [25].

Despite advances in measles vaccination, which helped to prevent about 60.3 million deaths from 2000 to 2023, global vaccination coverage declined during the COVID-19 pandemic [26]. Coverage with the first dose of measles vaccine (MCV1) dropped to 81% in 2020, the lowest level since 2008, before improving to 83% in 2022, unchanged in 2023. This stagnation in vaccination coverage has left millions of children unprotected, contributing to the increase in cases and outbreaks globally. In 2025, there was a dramatic increase in measles cases, primarily in the United States, Canada and Mexico. 8,839 confirmed measles cases and 13 related deaths were reported in the Region of the Americas between Epidemiological Week (EW) 1 and EW 28 in 2025 [27] (Fig. 1b). The most affected countries were Canada, with 3,969 cases and one death; Mexico, with 3,361 cases and nine deaths; and the United States, with 1,308 cases and three deaths. The North American measles epidemic that began this January in Texas has spread through a network of undervaccinated religious communities into Canada and Mexico, threatening the official measles-free status of all three countries [25]. Notably, these numbers are provisional, reflecting data at the time of submission, as the situation is rapidly evolving.

#### Canada

**Fig. 2:**
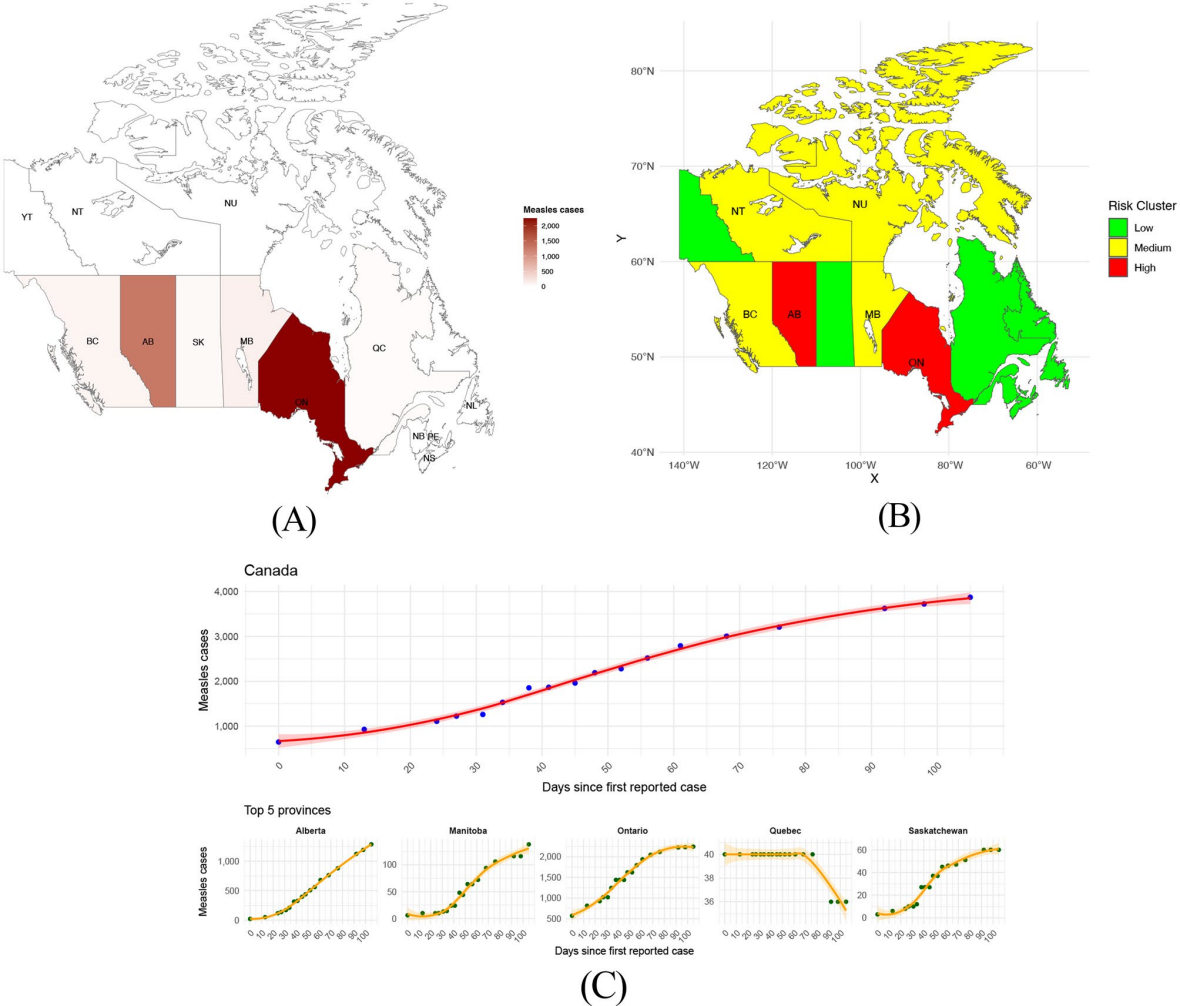
**A**. Geographic distribution of confirmed measles cases by province in Canada from March 30 to July 13, 2025. **B**. Spatial distribution of modeled relative measles risk across Canadian provinces. **C**. Epidemic trajectories for selected provinces. Red lines represent epidemic growth curves estimated through log-linear regression models, while pink shaded areas indicate the 95% confidence intervals. Daily growth rates were derived from the slope coefficients of these models applied to quasi-daily case counts. The time period extends from the first reported case (end of March 2025) to day 105 (mid-July 2025)

Canada is currently facing its largest measles outbreak in recent history, with transmission patterns varying widely across provinces. Ontario (2,244 cases) and Alberta (1,284 cases) were the most affected provinces (Fig. 2a). Spatial modeling of measles risk revealed marked geographic heterogeneity across Canada (Fig. 2b), with a mean national relative risk of 4.64, a median of 1.03, a minimum of 0.02, and a maximum of 31.56. Some regions, particularly Ontario, showed extremely high risk levels, with maximum values exceeding 31 and an average provincial risk score of 31.6. Alberta followed with an average risk of 18.0, while provinces such as Manitoba (1.94) and British Columbia (1.47) showed moderate risk levels. Northern territories, such as the Northwest Territories and Nunavut, had average risk scores of around 2, despite low case numbers, suggesting spatial spread and local vulnerability. Quebec and the Atlantic provinces, including Newfoundland and Labrador, New Brunswick, and Nova Scotia, maintained lower risk profiles, with averages below 0.7.

Epidemic dynamics showed significant variations between provinces, as illustrated in Fig. 2c. Alberta experienced the fastest epidemic growth with an average daily increase of 31.7%, reaching a peak of 1,284 cases on day 105, accompanied by high variability ($\omega$ = 399) and a significant cumulative burden (AUC = 9,158 case-days). British Columbia showed a similar growth rate (34.0% daily) but with a more contained epidemic (peak of 104 cases, AUC = 374). Manitoba had moderate growth of 23.4% daily and a peak of 138 cases, while Ontario, despite a slower growth rate (8.84% daily), it sustained the highest epidemic burden, with a peak of 2,244 cases and an AUC of 27,754 case-days, reflecting prolonged transmission and high variability ($\omega$ = 529). Quebec showed a slight daily decrease (-0.588%), consistent with controlled transmission and a modest peak of 40 cases. Saskatchewan had a peak of 60 cases on day 92, with moderate variability ($\omega$ = 20.1) and an AUC of 573. Complete metrics on provincial risk and epidemic characteristics are shown in Table 1. Ontario and Alberta fall into the high-risk cluster, with average values of 31.6 and 18.1, respectively, while Manitoba, Nunavut, British Columbia, and the Northwest Territories show moderate risks (1.47–2.59). The other provinces maintain low risk profiles, consistent with the low number of cases and the absence of large outbreaks. Overall, the model shows a very good fit to the observed data across all provinces, with an R$^2$ $\approx$ 0.979.

**Table 1:** Summary of key epidemiological parameters by province in Canada

**Province**	**Daily growth (%)**	**Peak day**	**Cases at peak**	**Variability** ($\omega$)	**AUC (case-days)**	**Outbreak duration (days)**	**Interpretation**
Ontario	8.84	105	2,244	529	27,754	106	Prolonged epidemic with high peak, high pressure on the healthcare system.
Alberta	31.7	105	1,284	399	9,158	106	Rapid growth, consistent load over time, moderate peak compared to Ontario.
British Columbia	34.0	105	104	30.3	374	106	Contained and stable epidemic, short duration.
Manitoba	23.4	105	138	44.0	973	106	Epidemic moderate in both intensity and duration.
Quebec	-0.588	0	40	1.53	708	106	Controlled transmission, low peak, low variability.
Saskatchewan	23.1	92	60	20.1	573	106	Short and moderate epidemic, limited peak.

From a demographic perspective, the epidemic in Canada predominantly affected unvaccinated individuals, who accounted for 86% of cases. The majority of infections occurred in children, with 45% of cases in those aged 5 to 17 years and 20% in children aged 1 to 4 years. Males represented 51% of the total cases, while females accounted for 49%. Vaccination coverage was notably low, with only 2% of affected individuals having received one dose and 5% having received two or more doses. The epidemic also impacted a small proportion of older adults, with 1% of cases observed in individuals aged 55 and above.

To evaluate whether the distribution of cases was significantly associated with vaccination status, a chi-squared test ($\chi^2$) was performed on the contingency table that compared the number of cases across different vaccination groups (Unvaccinated, 1 dose, 2+ doses, Unknown). The results of the test showed a highly significant association between vaccination status and the number of cases ($\chi^2$ = 4984, df = 3, p-value $<$ 2.2e-16), indicating that the distribution of cases across vaccination groups was not due to random chance. Specifically, the test revealed that unvaccinated individuals were disproportionately affected by the outbreak, accounting for the majority of reported cases. A post-hoc analysis using Fisher’s Exact Test was performed to further examine the relationship between the unvaccinated and vaccinated groups. The result of the Fisher’s test (p-value = 7.43e-06) confirmed that the odds of infection were significantly higher in the unvaccinated group compared to the vaccinated individuals.

#### Mexico

**Fig. 3:**
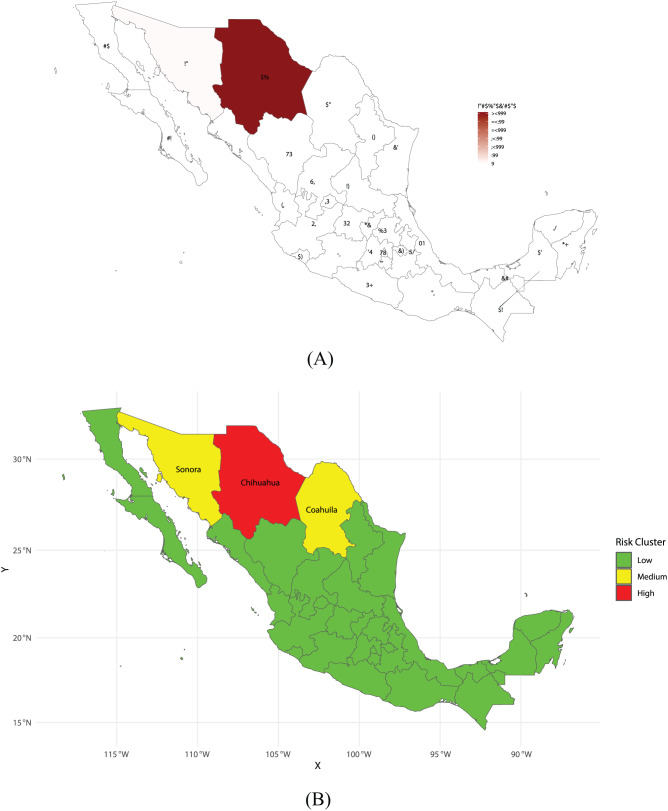

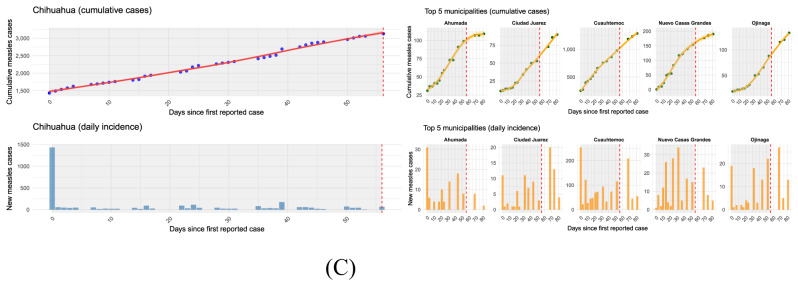
**A** Geographic distribution of confirmed measles cases by municipalities in Mexico from May 19 to July 14, 2025. **B** Distribution of modeled measles risk across municipalities. **C** Epidemic curves for selected municipalities of Chihuahua, showing both cumulative cases (top row) and daily incidence (bottom row). Shaded grey areas indicate the observed surveillance period (days 0–56, corresponding to May 19 to July 14, 2025). Red dashed vertical lines mark the transition between observed data and model simulations (days 57–80). Beyond day 56, the curves represent projections generated by the model, allowing the detection of possible late peaks (e.g., Ojinaga peaking at day 68)

Mexico is currently facing its largest measles outbreak in decades, centered in the Mennonite community of Cuauhtémoc, Chihuahua, with 3,129 cases and 8 deaths reported. Women accounted for 52% of confirmed cases, while men represented 48%. The most affected age group was children aged 0–4 years (709 cases, 6.82% per 100,000 inhabitants), followed by young adults aged 25–29 years (474 cases, 4.47% per 100,000 inhabitants) and 30–34 years (401 cases, 3.84% per 100,000 inhabitants). Vaccination coverage among cases was notably low, with 92.4% of confirmed patients having no documented doses of MMR vaccine. An overview of the key epidemiological parameters for the most affected municipalities is summarized in Table 2. Spatial modeling of relative measles risk across Mexican municipalities (Figu. 3b) revealed a highly skewed distribution of risk, with a national mean of 9.47, a median of 0.43, and a standard deviation of 49.1. Risk scores ranged from 0.12 to 283, underscoring stark disparities in vulnerability. Chihuahua stands out with an extreme risk value (mean and median: 283), placing it in the high-risk cluster, while Sonora and Coahuila were categorized as medium-risk states with scores of 7.49 and 3.57, respectively. The epidemic trajectories in the main affected municipalities (Fig. 3) reveal differing temporal patterns but overall show a very good fit of the model to observed data ($R^{2} \approx 0.84$). Specifically, Fig. 3 highlights that Chihuahua experienced a sustained increase in cases, peaking at 106 new cases on day 68 with an average growth rate of about $2.76 \times 10^{-7}$. Cuauhtémoc displayed a pronounced epidemic trajectory, with an initial cluster of 254 cases recorded at day 0 (May 19, 2025), corresponding to the baseline level reported at the start of the surveillance period. These cases do not represent imported infections, but rather mark the beginning of local transmission in the municipality. Cuauhtémoc also showed the highest variability among the municipalities examined ($\omega \approx 66.7$) and the largest cumulative burden (AUC $\approx$ 1,132). In contrast, Nuevo Casas Grandes exhibited a later peak on day 32 with 34 new cases and the highest growth rate ($6.51 \times 10^{-7}$), although with a more moderate overall burden (AUC $\approx$ 187) and intermediate variability ($\omega \approx 10.7$). Other municipalities, such as Ahumada, Ojinaga, and Riva Palacio, displayed more modest epidemic dynamics. Ahumada peaked early at day 0 with 31 cases, low variability ($\omega \approx 8.5$), and an AUC of about 92.5. Ojinaga peaked later, on day 68, with 27 new cases, low variability ($\omega \approx 9.0$), and an AUC near 117. Finally, Riva Palacio showed the smallest cumulative burden (AUC $\approx$ 41), with a peak at day 0 of 40 cases and low variability ($\omega \approx 10.1$).

**Fig. 4:**
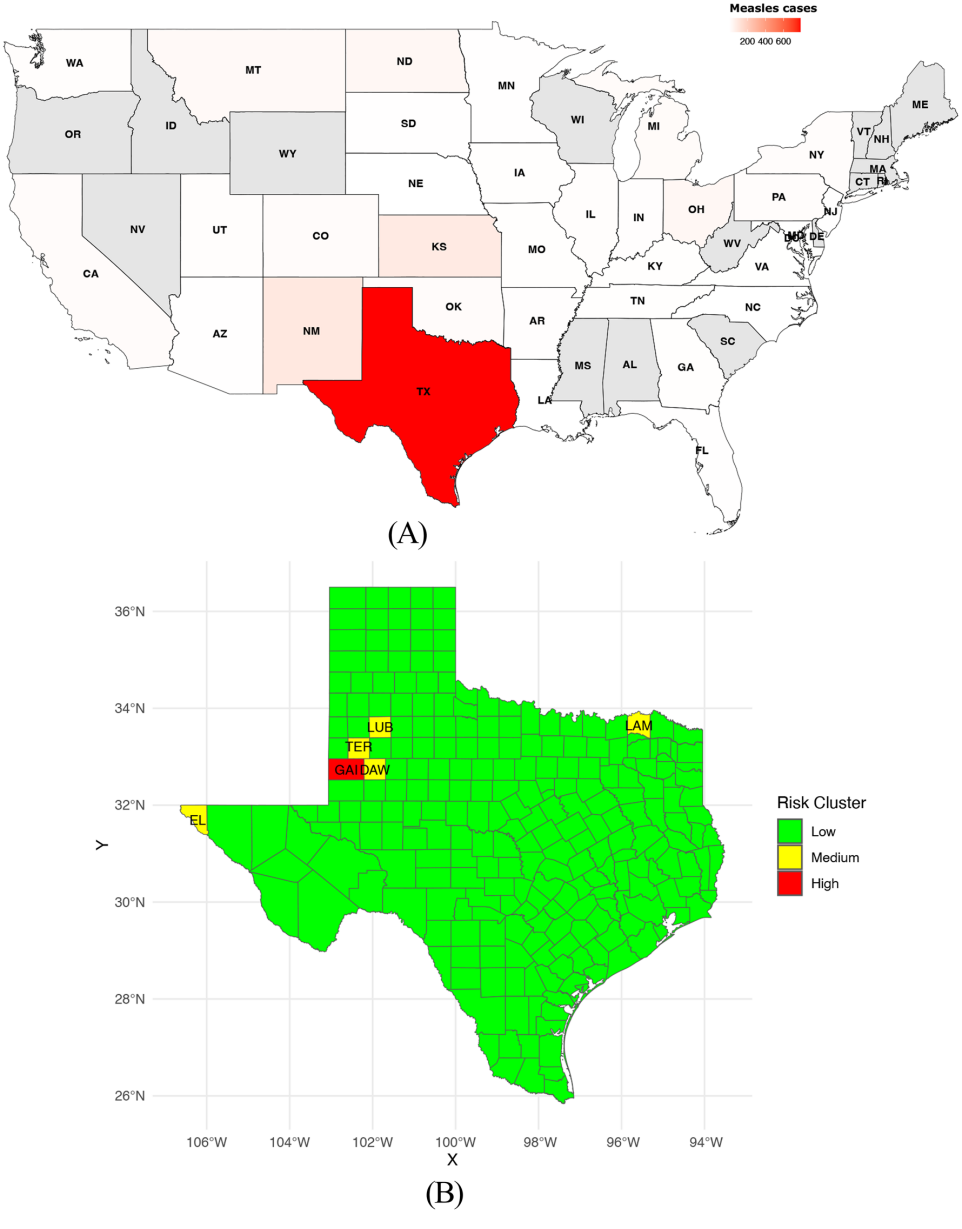

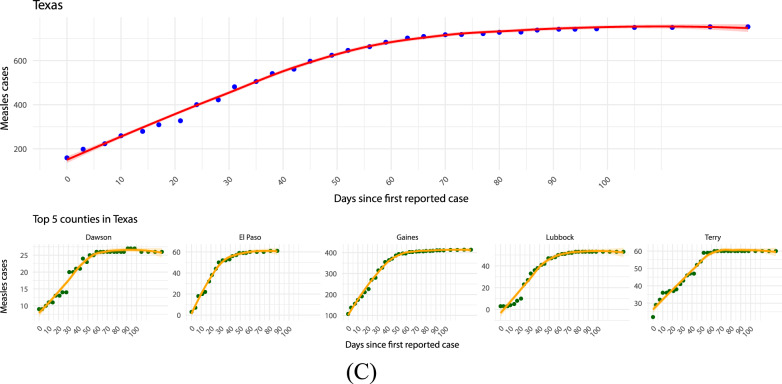
**A** Geographic distribution of confirmed measles cases by state in USA from March 4 to July 22, 2025. **B** Spatial map of estimated relative measles risk across Texas counties. **C** Epidemic trends for selected counties

**Table 2:** Summary of key epidemiological parameters by selected high- and medium-risk municipalities in Mexico

**Municipality**	**Daily growth (%)**	**Peak day**	**Cases at peak**	**Variability** ($\omega$)	**AUC (case-days)**	**Outbreak duration (days)**	**Interpretation**
Ahumada	9.75	81	109	29.0	1,005	82	Moderate outbreak, early peak, low cumulative burden.
Nuevo Casas Grandes	85.7	81	190	67.8	1,368	82	Rapid increase, moderate cumulative burden, intermediate variability.
Cuauhtémoc	12.7	81	1,286	340	10,675	82	Severe outbreak, very high peak and cumulative burden, highest variability.
Ciudad Juarez	17.0	81	90	27.6	554	82	Moderate outbreak with early peak, low cumulative burden.
Ojinaga	15.8	81	133	40.5	818	82	Moderate outbreak, delayed peak, intermediate cumulative burden.
Riva Palacio	1.02	0	40	10.1	41	82	Small outbreak, low peak, low variability and cumulative burden.

#### United States

In 2025, the United States experienced its most severe measles resurgence since the official elimination of the disease in 2000, recording the highest case counts in more than three decades, distributed across 37 jurisdictions (Fig. 4a). The majority of cases (89%) were linked to outbreaks, with a particularly large and sustained outbreak spanning the states of Texas, New Mexico, and Oklahoma, accounting for 69% of all reported infections. The age distribution revealed that children under 20 years represented over 60% of cases, highlighting the vulnerability of younger populations. Vaccination history data indicated that 95% of cases were either unvaccinated or had unknown vaccination status, underscoring gaps in immunity and the critical role of vaccine coverage. Hospitalizations occurred in 12% of cases, with young children especially at risk of severe disease. Notably, MMR vaccination coverage among children has shown a slight but concerning decline in recent years, from 95.2% in 2019–2020 to 92.7% in 2023–2024, potentially contributing to increased susceptibility.

Spatial risk modeling focusing on Texas (Fig. 4b) revealed a highly heterogeneous and skewed distribution of relative measles risk across counties, with a mean risk score of 45.3 and a median of 1.04. Most counties fell within a low-risk cluster, while a small subset exhibited exceptionally high risk scores, concentrated primarily in the northwestern and central regions of the state. This spatial clustering highlights focal areas of intense transmission and ongoing outbreak activity. Five counties identified as having the highest inferred risk—Gaines, Terry, Lubbock, El Paso, and Dawson—were selected for detailed temporal epidemic analysis (Fig. 4c). Each county exhibited distinct transmission dynamics but all showed excellent model fits to observed case data (R$^2$ $\approx$ 0.997), reflecting robust epidemic characterization. At the state level, Texas saw sustained epidemic growth with an average daily increase of 1.23%, reaching its peak on day 119. The epidemic was marked by high day-to-day variability and a substantial cumulative burden measured by the AUC.

**Fig. 5:**
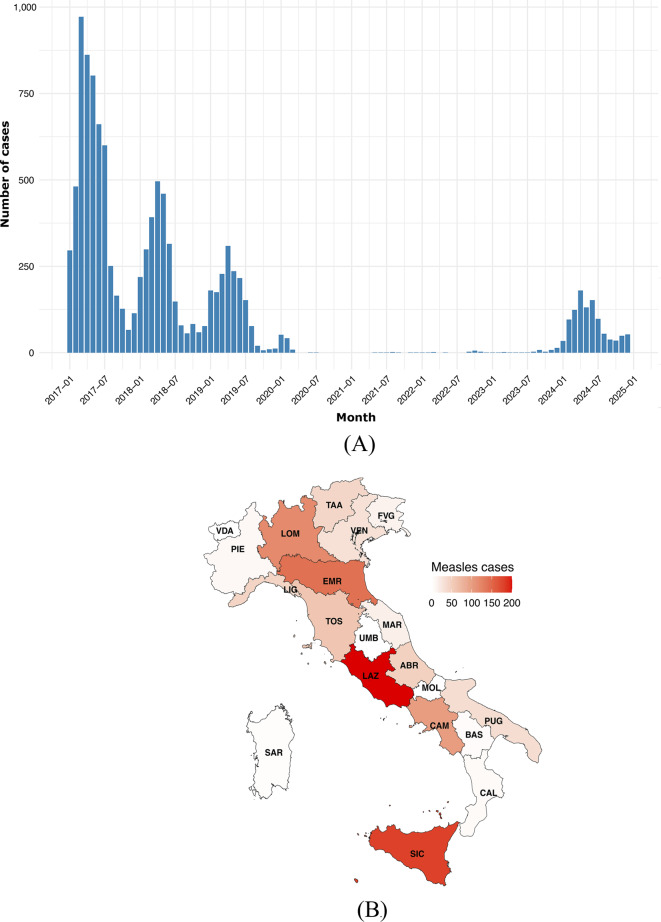

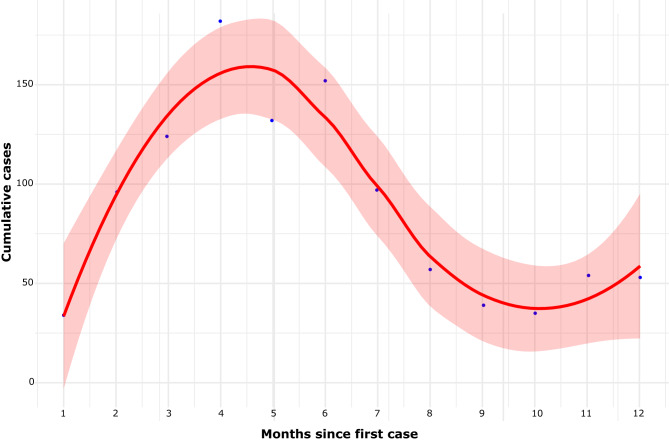
**A** Temporal distribution of measles cases in Italy, 2017–2025 (as of June 30, 2025). **B** Geographic distribution of measles cases by region in 2024. **C** Epidemiological dynamics of measles in Italy (monthly scale), 2024

The epidemiological dynamics of the five selected counties are summarized in Table 3. Gaines County recorded the largest outbreak, with 414 peak cases and the highest cumulative burden (AUC = 11,191), reflecting intense and prolonged transmission. El Paso County showed the fastest epidemic expansion, with a daily growth rate of 20.3% and an earlier peak (day 81), although with a smaller cumulative burden compared to Gaines. Dawson County had the lowest number of peak cases (27) and the smallest overall epidemic burden (AUC = 702), consistent with a limited outbreak. Lubbock and Terry Counties experienced intermediate outbreaks, with daily growth rates of 11.3% and 3.36%, respectively, and moderate epidemic burdens. Together, these heterogeneous epidemic profiles underscore the patchy nature of measles transmission across Texas, shaped by local vaccination gaps and community-level susceptibility.

**Table 3:** Summary of key epidemiological parameters for selected high-risk counties in Texas, USA (2025)

**County**	**Daily growth (%)**	**Peak day**	**Cases at peak**	**Variability** ($\omega$)	**AUC (case-days)**	**Outbreak duration (days)**	**Interpretation**
Dawson	3.68	91	27	6.40	702	127	Small outbreak, short peak, limited cumulative burden and low variability.
El Paso	20.3	81	61	18.9	983	89	Rapid outbreak, high growth rate but moderate overall burden.
Gaines	4.51	119	414	97.2	11,191	127	Intense and prolonged outbreak with very high peak and cumulative burden.
Lubbock	11.3	77	53	19.1	1,257	127	Moderate outbreak with intermediate peak and duration.
Terry	3.36	63	60	11.6	1,674	127	Moderate outbreak, early peak, intermediate cumulative burden.

### Epidemiological overview of measles outbreaks in Italy

Italy experienced a sharp increase in measles cases in 2024, with 1,055 confirmed infections, a dramatic rise compared to the 44 cases reported in 2023 (Fig. 5a). The outbreak predominantly affected unvaccinated individuals, who accounted for over 90% of cases. Of those infected, 50% required hospitalization, with severe complications including pneumonia and encephalitis reported in about 35% of patients. The majority of cases were concentrated in the Lazio, Sicily, Emilia-Romagna, Campania, and Lombardy regions (Fig. 5b), with a significant number occurring in densely populated urban areas, which may reflect higher transmission potential.

**Fig. 6:**
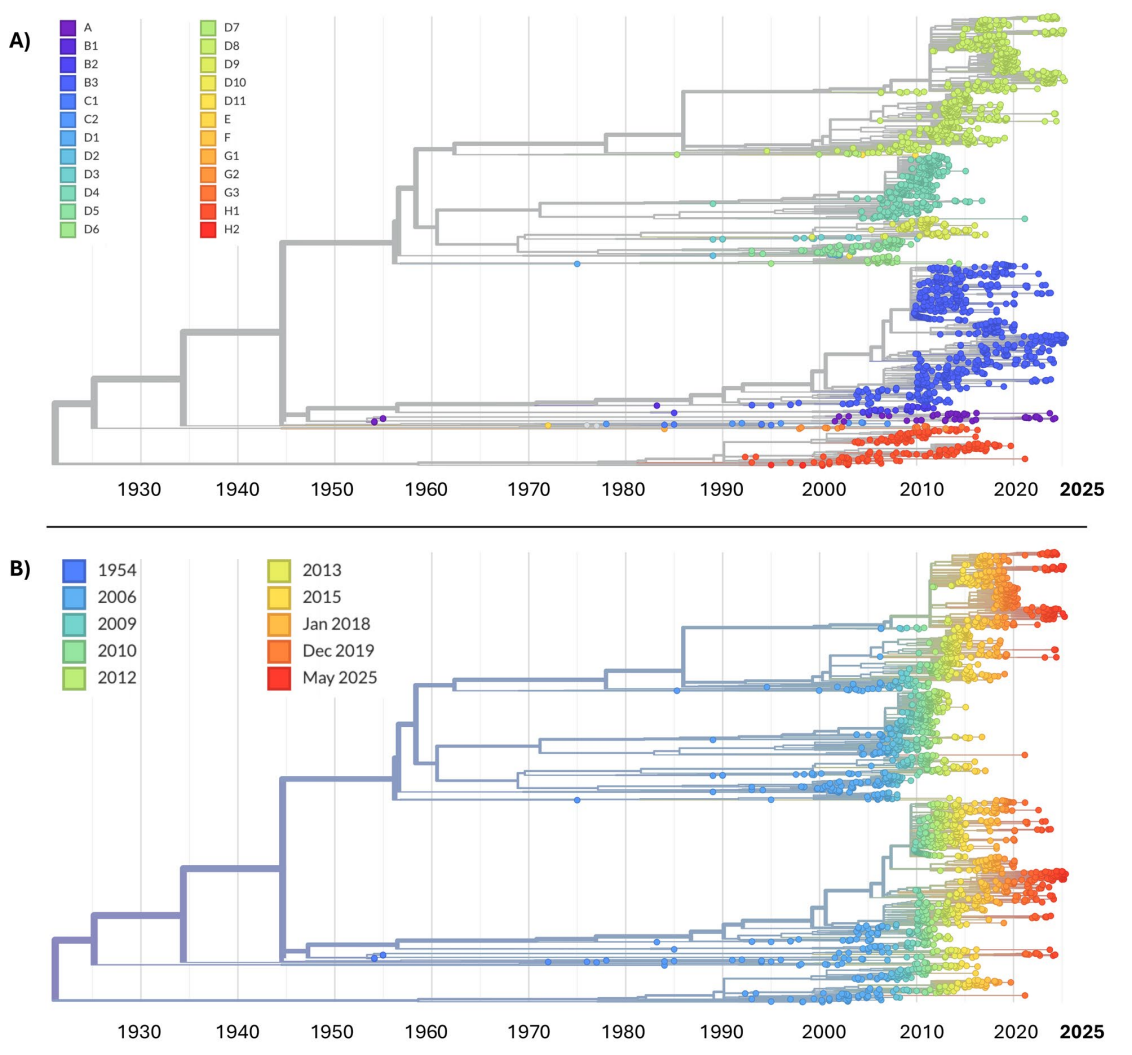
Time-oriented phylogenomic tree built on 2,353 complete genomes of measles (collection date: January 1954 - May 2025). **A** Isolates labeled according to the genotype of belonging. **B** Isolates labeled according to years of collection. The final figure was graphically refined using GIMP version 2.8 (https://www.gimp.org/downloads/oldstable/, accessed 8 July 2025)

Analyzing the various phases of the 2024 epidemic in Italy, as shown in Fig. 5c, reveals distinct differences in growth patterns. In the initial stage (January–February 2024), we estimated the growth rate using a log-linear regression model on the monthly case counts. The growth coefficient was 1.04 (95% CI: 0.65–1.43; p = 0.002), corresponding to a monthly exponential increase of about 182% (95% CI: 91–318%). This sharp rise is consistent with the typical start-up phase of an epidemic, although the wide confidence interval reflects the limited number of observations and the aggregation of data at monthly resolution. During the intermediate phase (March–May 2024), a quasi-Poisson log-linear regression yielded a slope of 0.024 (95% CI: –0.386–0.434), corresponding to a non-significant monthly change of +2.4% (95% CI: –32.0% to +54.4%). This suggests that, after the initial surge, the epidemic entered a more stable phase without clear growth or decline. Finally, in the later stage (June–December 2024), the estimated slope was –0.225 (95% CI: –0.367– –0.083), corresponding to a statistically significant monthly decline of –20.2% (95% CI: –30.7% to –8.0%). This indicates a phase of progressive containment, consistent with the reduction in reported cases.

Demographically, the outbreak affected both children and young adults, with a significant portion of cases involving individuals aged between 15 and 39 years. A notable concern was the infection of healthcare workers, who represented nearly 8% of reported cases, raising alarms about nosocomial transmission and the need for stricter immunization requirements in medical settings. Additionally, approximately 16% of cases occurred in children under five years old, emphasizing the heightened risk for severe disease in this vulnerable age group.

The resurgence of measles in Italy has been attributed to multiple factors, including a decline in routine childhood immunization rates, with the national measles vaccination coverage at 94% for the first dose and 90.3% for the second dose in children aged two years. These levels remain below the 95% threshold recommended by the World Health Organization for herd immunity. We used logistic regression models to assess the distribution of measles cases across three groups: individuals vaccinated with the first dose, those vaccinated with two doses, and the unvaccinated. A chi-squared ($\chi^2$) test of independence was conducted to determine if there was a significant association between vaccination status and the occurrence of measles cases. The test resulted in an extremely high chi-squared statistic ($\chi^2$ = 10,176, df = 2, p-value $<$ 2.2 $\times$ 10$^{-16}$), providing strong evidence to reject the null hypothesis of an equal distribution. This finding suggests that the distribution of measles cases is not uniform across vaccination groups, with unvaccinated individuals contributing disproportionately to the majority of cases.

To contextualize the Italian outbreak within the broader international scenario, we compared its key epidemiological indicators with those of the most recent measles epidemics in the United States, Canada, and Mexico (Table 4). This comparative overview highlights both common features, such as the strong association with lack of vaccination, and distinctive characteristics, such as the exceptionally high proportion of hospitalizations observed in Italy.

**Table 4:** Epidemiological summary of measles outbreaks (2024–2025) by country

**Country**	**Cases**	**Deaths**	**Vaccination status of cases**	**Age distribution of cases**	**Severity indicators**
United States (EW 1–28, 2025)	1,308	3	95% unvaccinated or unknown	$>$60% under 20 years	3 deaths; 12% hospitalized
Canada (EW 1–28, 2025)	3,969	1	86% unvaccinated; 7% vaccinated (1 or 2+ doses)	45% aged 5–17; 20% aged 1–4	1 death; 8 congenital cases; 48 cases in pregnant women (81% unvaccinated)
Mexico (EW 1–28, 2025)	3,361	9	92.4% unvaccinated	Most affected: 0–4 yrs, 25–34 yrs	9 deaths
Italy (2024 outbreak)	1,055	0	90% unvaccinated	16% under 5; majority 15–39 yrs; 8% healthcare workers	50% hospitalized; 35% severe complications

### Genetic analyses

The phylogenomic tree (Fig. 6) reveals clear genetic and temporal structuring. The phylogenomic reconstruction shows two representations of the same analysis: one with terminals labeled according to genotype (A) and one according to isolation date (B). The most striking result is the genotypic diversity over time. Indeed, Fig. 6a shows a clear separation between major genotypic clades, reflecting the historical diversification of the virus. Some genotypes, particularly within group D (e.g., D1–D11), appear highly branched and represent the bulk of recent viral diversity, suggesting substantial evolutionary persistence and widespread circulation. In contrast, earlier genotypes such as A and B are phylogenetically isolated and temporally restricted (1950s–1970s), implying they represent evolutionary dead-end lineages with eventual extinction or drastic reduction. Clades D4, D8, and H1 appear dominant. Their long branches suggest considerable intra-genotypic diversity, likely resulting from sustained transmission and geographic diversification within those genotypes. Figure 6b depicts the chronological view of the same evolutionary structure. It is interesting to note the progressive accumulation of genetic diversity over time, with the majority of sequences clustering from the 1990s to 2025, is consistent with the global expansion of molecular surveillance. The continuous temporal gradient—from the earliest samples (blue) to the most recent (red)—suggests that genetic divergence has increased steadily over time, with long, complex terminal branches in more recent clades indicating ongoing molecular evolution. The overlap between recent strains and dominant genotypes (e.g., D8, H1) suggests that these represent the most actively circulating strains in recent decades.

**Fig. 7:**
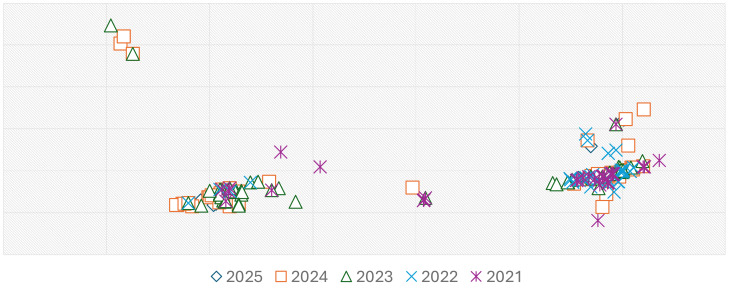
Principal Coordinates Analysis (PCoA) of the N450 region of Measles, with sample groups defined a priori according to the collection year. Each symbol represents a single sequence plotted as a point corresponding to its respective year. The axes represent the principal coordinates that explain the highest proportion of variance among the sequences. The two-dimensional projection (PCoA1 and PCoA2) illustrates genetic divergence among the sequences, attributable to nucleotide differences per site within the dataset. The first three axes account for a total of 95.21% of the observed genetic variation (Axis 1: 89.68%; Axis 2: 3.90%; Axis 3: 1.62%)

Tree topology also indicates genotype replacement events, with some clades emerging and disappearing rapidly, while others expand and persist, suggesting possible selective advantages, introductions, or changes in population immunity pressure. This scenario is likely influenced by vaccination. Overall, the observed genetic and temporal variability is consistent with a complex epidemiological dynamic characterized by co-circulation of multiple genotypes, clonal expansions, and potential bottlenecks related to vaccination campaigns or mobility restrictions. Certain clades show patterns of local persistence followed by global spread, supporting the hypothesis that regionally confined strains can become dominant on a wider scale. Moreover, the absence of recent sequences from historically recognized genotypes (e.g., B1–B3, C1–C2, G1–G3) aligns with epidemiological data reporting the progressive elimination of multiple genotypes following widespread vaccination efforts. The dominance of a few surviving genotypes reflects a possible evolutionary bottleneck driven by population-level immunity. The PCoA plot (Fig. 7) sheds light on more recent differentiation (2021–2025). The plot reveals low temporal structuring across samples collected during this period. Although minor clustering patterns can be observed, especially among samples from 2021 and 2022 (right side of the plot), and a smaller group including sequences from 2024 and 2025 (left side), the overall distribution suggests substantial overlap across years. No clear separation along the first two PCoA axes is evident, indicating that genetic variation does not strongly correlate with sampling year over the examined timeframe.

**Fig. 8:**
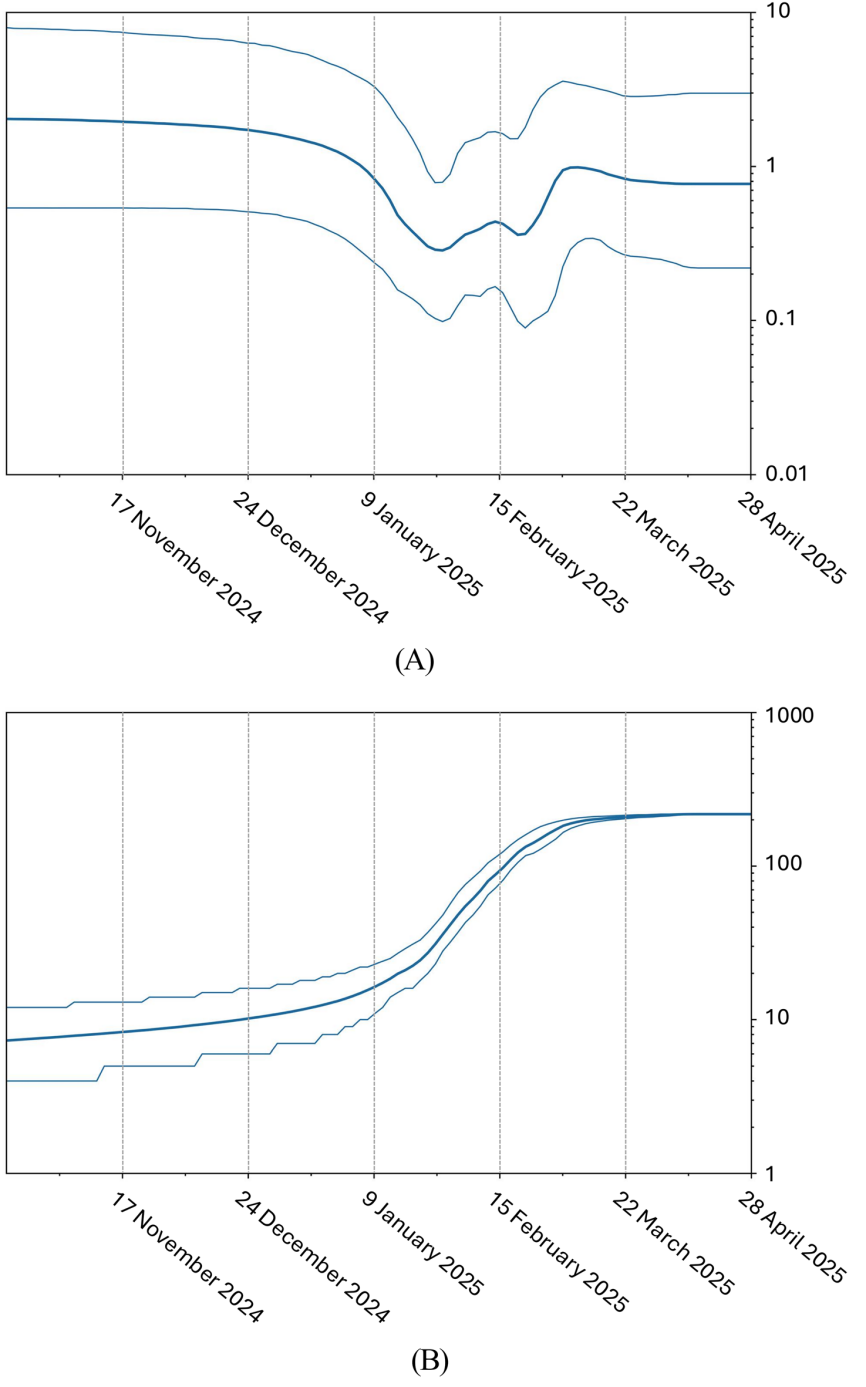
**A** Bayesian Skyline Plot (BSP) of Measles N450 region. The effective population size is presented on the y-axis, plotted over time along the x-axis. **B** Lineages Through Time (LTT) of Measles N450 region. The number of lineages are presented on the y-axis, plotted over time along the x-axis. The three lines represent the median value (thick central line) and the corresponding confidence interval (upper and lower thin lines, indicating the maximum and minimum values of the interval, respectively). The visual representation was post-processed using GIMP software, version 2.8 (accessible at https://www.gimp.org/downloads/oldstable/, last accessed on 9 July 2025)

This pattern suggests multiple lineages have been co-circulating concurrently during this five-year period, possibly reflecting either sustained transmission chains or repeated introductions of genetically similar viruses. The relative dispersion of points along both axes, combined with the absence of compact temporal clusters, further supports ongoing genetic mixing rather than strict temporal succession or lineage replacement. These findings are consistent with the phylogenetic patterns observed in the N450 trees, which highlight the persistence and overlapping circulation of specific genotypes over time. The two profiles differ not only in specific nucleotide positions but also in the degree of variability. Genetic variability appears higher in 2024, whereas the 2025 sequences seem more stable. While this may be partly attributable to the smaller number of sequences available for 2025, the difference persists even when the data are normalized to the minimum number of sequences in the 2025 dataset, suggesting an inherently more conserved profile compared to 2024. The phylodynamic analysis based on N450 region sequences of the measles virus genome revealed a pattern consistent with a recent epidemic expansion. The Bayesian Skyline Plot (BSP) (Fig. 8a) shows a marked increase in the effective population size (Ne) in the recent past, indicating intense viral transmission. This pattern is consistent with the occurrence of the outbreaks reported in Italy (2024) and Texas (2025). Such an increase suggests a surge in secondary infections over a relatively short time frame, likely facilitated by suboptimal vaccination coverage falling below the critical herd immunity threshold. Likewise, the Lineages Through Time (LTT) (Fig. 8b) plot indicates a progressive increase in the number of evolutionary lineages over time, suggesting rising genetic diversity within the viral population. This pattern is indicative of sustained transmission and possibly the concurrent circulation of multiple viral lineages, supported by phylogenetic tree structure and the observed dispersion in the PCoA. Together, these findings reflect not only a numerical expansion of the viral population but also increased genetic complexity, likely driven by multiple introductions and/or prolonged transmission in under-vaccinated settings.

From an epidemiological and evolutionary perspective, these dynamics are consistent with a scenario in which declining population-level immunity—due to delays in vaccination, incomplete coverage, or spatial heterogeneity—enables the virus not only to spread more efficiently but also to maintain a broader genetic pool. This, in turn, can favor the persistence and dissemination of specific genotypes (such as D8 and H1, highlighted in the phylogenetic analysis), which act as markers of epidemic phases. While transmission is ultimately driven by phenotypic traits, these traits are encoded by the underlying genotype, so advantageous phenotypes indirectly promote the spread of the corresponding genotypes. However, there is currently no evidence of specific adaptive selection or functional change associated with this increased diversity; the observed pattern appears to be primarily demographic (i.e., reflecting population expansion) rather than directional molecular evolution. Finally, the temporal overlap of multiple viral lineages suggests that both external reintroductions and local transmission may have played a role. This underlines the importance not only of restoring and maintaining high vaccine coverage but also of reinforcing molecular surveillance systems [28] to detect early signals of re-emergence and to monitor viral genetic diversity over time.

## Conclusions

Despite occurring in markedly different contexts, the 2024 Italian and 2025 Americas measles outbreaks both underscore the pivotal importance of vaccination in limiting transmission and safeguarding public health in the context of highly contagious diseases such as measles [29]. From an epidemiological perspective, Italy experienced a slower but more sustained increase in measles incidence, with the number of cases progressively rising over several months. However, the availability of data only on a monthly aggregate scale severely limited the granularity of the analysis, preventing the accurate detection of short-term fluctuations or sharp spikes in transmission. In contrast, the epidemic in the Americas, plotted at near-daily resolution, allowed an high-resolution epidemiological snapshot with an advanced spatial analysis and dynamic risk modeling, using the a bayesian spatial modeling approach. By identifying localized outbreaks with high risk, this approach enables targeted immunization, communication, and containment interventions. Vaccination status emerged as a key determinant in both outbreaks. For example, in Canada and Italy, statistical models confirmed a clear association between immunisation and reduced incidence, highlighting the importance of having such granular data. In the case of Mexico and Texas, however, we were unable to find such information, which limited our ability to analyse the effectiveness of immunisation policies. Some limitations need to be considered. First, the analysis is based on aggregate data, without access to individual information (e.g., comorbidities, socioeconomic status), which could improve understanding of the determinants of transmission. Second, the quality of surveillance data is not uniform across countries in the region and may be affected by under-notification or delays in reporting. Third, while offering a highly detailed picture of spatial risk, the modeling used does not incorporate behavioral or local health context variables that could further enrich prediction and epidemiological interpretation. Ultimately, this study underscores how successful elimination is not a definitive goal, but a fragile condition to be maintained with ongoing, evidence-based efforts, international collaboration, and investment in resilient health infrastructure. Strengthening vaccine confidence, rebuilding collective immunity, and ensuring equity in access to preventive services are imperative priorities for the future of global public health.

The genetic analysis reveals a pronounced reduction in genetic diversity from Italy to Texas, suggesting the occurrence of a genetically diverse viral population in Italy to a much more conserved one in Texas. This differences in genetic variablity levels can be explained by an interplay of purifying selection, which removes suboptimal variants to maintain functional optimization, and founder effect, where only a small subset of viral genomes seed new transmission chains and carry forward limited diversity. The Texan lineage’s conservation may reflect fixation of variants that optimize chaperone-coordinated polymerase tethering by stabilizing the NTAIL motifs that anchor the polymerase complex via Hsp70/Hsp40 cochaperones, supporting efficient replication in close-contact settings. Similarly, selection for optimized ribonucleocapsid assembly geometry could balance particle stability and uncoating efficiency under specific epidemiological pressures. The observed conservation might also reflect selection for enhanced evasion of RIG-I-like receptor sensing by maintaining interfaces that suppress interferon responses more effectively, as well as fixation of T-cell escape mutations capable of sustaining transmission in communities with patchy adult immunity. In addition, epistatic coevolution with other viral proteins, including matrix and polymerase components, likely constrained variability to preserve functional replication complexes, while modulation of inclusion-body biophysics through conserved NTAIL sequences may have optimized replication compartment properties for community transmission. Temperature-sensitive replication dynamics may also have favored fixation of variants best suited to the local seasonal context. Finally, the role of founder effect and/or bottlenecks in respiratory droplet transmission cannot be overstated, as such phenomena dramatically reduce the variants complexity before selection further refines the viral population.

Overall, our findings underscore how epidemiological, social, and molecular factors interact to shape measles outbreak dynamics, highlighting the need to integrate genomic surveillance with traditional epidemiology to anticipate evolutionary trends, strengthen routine immunization, and maintain elimination goals in the face of evolving challenges. For what genetic analyses is concerned, our results highlight a dynamic landscape characterized by both long-term diversification and recent shifts in genetic complexity. The progressive increase in viral diversity observed between the 1990s and 2024 was followed by a notable contraction in 2025, putatively reflecting a combination of founder effect, purifying selection, and local transmission dynamics. Dominant genotypes such as D8 and H1 remain central to recent outbreaks, with signs of intragenotypic diversification in areas of sustained circulation. However, no evidence of adaptive selection or functional divergence was identified. This suggests that demographic and immunological factors - rather than viral evolution per se - primarily shape the epidemiology of measles in this phase. These findings reinforce the need for uninterrupted genomic surveillance, which remains essential not only for tracking viral spread and persistence [28], but also for anticipating possible shifts in genetic structure that could undermine current control strategies. Integrating high-resolution sequencing data with traditional epidemiological approaches will be pivotal to maintaining management goals in the face of evolving challenges.

## Data Availability

Epidemiological data presented in this study are available at: https://github.com/fbranda/measles (accessed on 22 July 2025). Genomic data were obtained from NCBI Virus (available at: https://www.ncbi.nlm.nih.gov/labs/virus/vssi/#/dashboard) (accessed on 8 July 2025).
